# Gadoxetic Acid-Enhanced T1 Mapping Enables Transporter-Mediated Molecular Imaging of Liver Functional Reserve

**DOI:** 10.3390/biomedicines14081695

**Published:** 2026-07-28

**Authors:** Yuting Zhu, Xun Hu, Zhuo Shi, Yuan Liang, Dengfeng Li, Peiqing Ma, Dong Yan, Jianwei Liang, Qian Wang

**Affiliations:** 1 Department of Imaging Diagnosis, National Cancer Center/National Clinical Research Center for Cancer/Cancer Hospital, Chinese Academy of Medical Sciences and Peking Union Medical College, Beijing 100021, China; 2 Department of Pathology, National Cancer Center/National Clinical Research Center for Cancer/Cancer Hospital, Chinese Academy of Medical Sciences and Peking Union Medical College, Beijing 100021, China; 3 Department of Interventional Therapy, National Cancer Center/National Clinical Research Center for Cancer/Cancer Hospital, Chinese Academy of Medical Sciences and Peking Union Medical College, Beijing 100021, China; 4 Department of Colorectal Surgery, National Cancer Center/National Clinical Research Center for Cancer/Cancer Hospital, Chinese Academy of Medical Sciences and Peking Union Medical College, Beijing 100021, China

**Keywords:** quantitative hepatobiliary MRI, Gd-EOB-DTPA, T1 mapping, liver functional reserve, transporter-mediated imaging, indocyanine green

## Abstract

**Aim:** To establish and validate a quantitative transporter-mediated imaging framework based on gadoxetic acid-enhanced T1 mapping for assessing liver functional reserve (LFR) and to investigate the physiological significance of the relative change in longitudinal relaxation rate (ΔR1%) as a quantitative imaging biomarker. **Methods:** Female C57BL/6J mice (6–8 weeks old) representing five experimental liver conditions (control, transporter-deficient *Slco1b2*/*Slco1a5* double-knockout, carbon tetrachloride-induced fibrosis, methionine–choline-deficient diet-induced steatohepatitis, and alcohol-associated fatty liver disease; n = 6 per group) underwent serial Gd-EOB-DTPA-enhanced T1 mapping. Quantitative ΔR1% was calculated to characterize hepatobiliary enhancement kinetics. Liver functional reserve was independently evaluated using multispectral optoacoustic tomography of indocyanine green (ICG) pharmacokinetics and serum ICG retention assays, with histopathological and hepatocellular transporter analyses performed for mechanistic validation. Longitudinal data were analyzed using restricted maximum likelihood (REML)-based mixed-effects models. Intergroup comparisons were performed using one-way ANOVA or Kruskal–Wallis tests with appropriate post hoc analyses, and associations between imaging and functional parameters were evaluated using Spearman rank correlation analysis. A two-sided *p* < 0.05 was considered statistically significant. **Results:** Five experimental liver models exhibited distinct transporter-dependent hepatobiliary enhancement patterns. The transporter-deficient knockout mice showed minimal enhancement, whereas fibrosis and steatotic liver injury models demonstrated intermediate but clearly distinguishable functional profiles. Longitudinal mixed-effects analysis identified significant effects of time, experimental group, and time-by-group interaction on ΔR1% dynamics (all *p* < 0.0001). Although MRI-derived ΔR1% parameters were not significantly correlated with regional optoacoustic ICG kinetics, ΔR1% area under the curve showed a strong inverse correlation with serum ICG retention at 600 s (r = −0.729, *p* < 0.0001), indicating that MRI-derived ΔR1% and ICG-based measurements provide complementary rather than interchangeable assessments of liver function. Histological and molecular analyses further demonstrated marked heterogeneity in fibrosis, steatosis, and hepatobiliary transporter expression across models, whereas transporter abundance alone did not consistently predict imaging-derived functional performance. **Conclusions:** Quantitative Gd-EOB-DTPA-enhanced T1 mapping provides a transporter-mediated imaging framework for evaluating hepatic functional reserve across mechanistically distinct liver injury models. As a normalized quantitative imaging biomarker, ΔR1% captures the integrated functional consequences of hepatobiliary transport dysfunction and complements established liver function tests. These findings support the translational potential of quantitative T1 mapping as a standardized, noninvasive approach for assessing liver functional reserve.

## 1. Introduction

Chronic liver disease (CLD) is a major global health burden characterized by progressive loss of liver functional reserve (LFR), ultimately leading to cirrhosis, hepatic decompensation, and hepatocellular carcinoma. Accurate assessment of LFR is therefore essential for disease staging, prognostic evaluation, perioperative risk stratification, and treatment planning [1,2,3,4,5,6]. Current clinical assessment primarily relies on composite scoring systems, such as the Child–Pugh classification, and systemic clearance tests including indocyanine green (ICG) retention. Although widely used, these approaches provide limited mechanistic information, lack spatial resolution, and may be insensitive to early functional impairment.

Hepatocyte-specific magnetic resonance imaging (MRI) using gadoxetic acid (Gd-EOB-DTPA) offers a unique opportunity to noninvasively evaluate hepatobiliary transport function [7,8,9,10]. Following hepatocellular uptake predominantly through organic anion transporting polypeptides (OATPs) and subsequent biliary excretion mediated by multidrug resistance-associated proteins (MRPs), Gd-EOB-DTPA enhancement reflects key physiological processes involved in liver function [11,12,13,14,15,16]. Consequently, hepatobiliary-phase MRI has become an increasingly important imaging tool for evaluating hepatic functional reserve.

In current clinical and experimental practice, however, Gd-EOB-DTPA-enhanced MRI is still predominantly interpreted using relative signal intensity measurements [17,18]. These measurements are highly dependent on acquisition parameters, receiver gain, field strength, and scanner-specific signal scaling, thereby limiting reproducibility and interstudy comparability [19,20,21]. Quantitative T1 mapping overcomes many of these limitations by directly measuring intrinsic tissue relaxation properties [22,23]. When combined with Gd-EOB-DTPA, quantitative T1 mapping enables calculation of the relative change in longitudinal relaxation rate (ΔR1%), providing a standardized quantitative metric of hepatobiliary contrast enhancement [24,25].

Although quantitative hepatobiliary MRI has attracted increasing interest, previous studies have largely focused on describing Gd-EOB-DTPA enhancement patterns or estimating liver function within individual disease entities, such as fibrosis or steatosis [26,27]. Most investigations have relied on single disease models or clinical cohorts and have primarily evaluated diagnostic performance rather than the biological mechanisms underlying quantitative enhancement. Consequently, it remains unclear whether ΔR1% represents a robust functional imaging biomarker that is applicable across different mechanisms of liver injury. In addition, the relationship between quantitative MRI measurements, hepatobiliary transporter expression, and established functional reference standards has not been systematically investigated [28,29,30,31,32].

To address these knowledge gaps, we systematically evaluated Gd-EOB-DTPA-enhanced quantitative T1 mapping across four complementary mouse models representing distinct mechanisms of hepatic dysfunction, including methionine–choline-deficient (MCD) diet-induced steatohepatitis, alcohol-associated fatty liver disease (AFLD), carbon tetrachloride (CCl_4_)-induced fibrosis, and a transporter-deficient *Slco1b2*/*Slco1a5* double-knockout model. Quantitative ΔR1% was further compared with systemic liver function assessed by serum ICG retention, regional hepatic ICG pharmacokinetics measured by multispectral optoacoustic tomography, and histological and molecular analyses of hepatobiliary transporter expression.

Unlike previous studies that primarily evaluated Gd-EOB-DTPA enhancement within individual liver diseases, the novelty of the present study lies in the multimodal mechanistic validation of quantitative T1 mapping across multiple mechanistically distinct liver injury models. By integrating quantitative MRI with functional, molecular, and histopathological analyses, we investigated whether ΔR1% reflects the integrated functional output of hepatobiliary transport rather than the abundance of individual transporters alone. This work establishes a mechanistic framework for interpreting quantitative Gd-EOB-DTPA-enhanced T1 mapping and supports its potential as a standardized, noninvasive imaging biomarker for assessing hepatic functional reserve.

## 2. Materials and Methods

Female wild-type C57BL/6J mice (6–8 weeks old; Beijing Vital River Laboratory Animal Technology Co., Ltd., Beijing, China) were housed under standard laboratory conditions (22 ± 2 °C, 50–60% humidity, 12 h light–dark cycle) with ad libitum access to food and water. Sample size (n = 6 per group) was determined based on previous longitudinal small-animal MRI studies to provide adequate statistical power for repeated-measures and intergroup comparisons.

Animals were randomly allocated using a computer-generated randomization schedule into four experimental groups: control, carbon tetrachloride (CCl_4_)-induced fibrosis, methionine–choline-deficient (MCD) diet-induced steatohepatitis, and alcohol-associated fatty liver disease (AFLD). Fibrosis was induced by intraperitoneal administration of 10% (*v*/*v*) CCl_4_ in olive oil (10 μL/g body weight) every three days for six weeks. Steatohepatitis was induced by feeding an MCD diet (TP3622, Nantong TeloPharm, Nantong, China) for six weeks, whereas AFLD was established using a Lieber–DeCarli liquid diet (TP4030B, Nantong TeloPharm, Nantong, China; 4% ethanol, approximately 28% of total caloric intake) for six weeks. All experimental procedures were performed at matched circadian time points to minimize physiological variability.

*Slco1b2*/*Slco1a5* double-knockout (KO) mice (C57BL/6J background; Shanghai Model Organisms Center, Shanghai, China) were included as a transporter-deficient functional reference group. Genotypes were confirmed by polymerase chain reaction (PCR) before enrollment. Detailed genotyping procedures and validation results are provided in the Supporting Information (Section S1, Table S1 and Figure S1).

To enhance the clinical relevance of this study, the selected animal models were chosen to represent major categories of liver dysfunction encountered in clinical practice, including transporter deficiency, hepatic fibrosis, metabolic steatohepatitis, and alcohol-associated liver disease. These models encompass distinct pathophysiological mechanisms leading to impaired hepatobiliary transport and declining liver functional reserve, thereby providing a clinically relevant framework for evaluating whether quantitative ΔR1% can serve as a robust imaging biomarker across heterogeneous liver diseases rather than a single disease entity.

All animal experiments were approved by the Ethics Committee for Laboratory Animals of the Cancer Hospital, Chinese Academy of Medical Sciences (Approval No. NCC2022A571) and were conducted in accordance with the ARRIVE guidelines.

### 2.1. MRI Acquisition and Quantitative ΔR1% Analysis

MRI examinations were performed using a 9.4 T small-animal MRI system (Bruker BioSpec, Ettlingen, Germany) equipped with a 26 mm volume coil. Mice were anesthetized with 1.5–2.0% isoflurane in oxygen. Body temperature (37.0 ± 0.5 °C) and respiratory rate were continuously monitored throughout imaging, and respiratory gating was applied to minimize motion artifacts. A 26-gauge tail vein catheter was placed for contrast administration.

Gd-EOB-DTPA (Primovist^®^, Bayer Healthcare, Leverkusen, Germany) was administered intravenously at 0.025 mmol/kg, followed by a 100 μL saline flush.

Quantitative T1 mapping was performed using a respiratory-gated variable repetition time (VTR) fast low-angle shot (FLASH) sequence. T1 values were calculated by voxel-wise nonlinear fitting of the longitudinal recovery model. Imaging parameters included a field of view of 30 × 30 mm^2^, matrix size of 256 × 256, slice thickness of 0.5 mm, and in-plane spatial resolution of 0.15 × 0.15 mm^2^. Detailed imaging parameters are summarized in Supporting Information Table S2.

Baseline T1 maps were acquired before contrast administration, followed by serial post-contrast acquisitions at 10, 20, 30, and 40 min using identical imaging settings. Liver regions of interest (ROIs) were manually delineated while excluding major vessels and bile ducts. ROI placement was performed in a blinded manner and propagated across all imaging time points for each animal.

The relative change in longitudinal relaxation rate (ΔR1%) was calculated as:

(1) $$\Delta R1\;(t,\%)\;=\;\frac{R1_{t}-R1_{0}}{R1_{0}}$$

where R1_0_ represents the pre-contrast longitudinal relaxation rate and R1_t_ represents the relaxation rate at each post-contrast time point.

### 2.2. Optoacoustic Imaging and ICG Pharmacokinetics

Hepatic indocyanine green (ICG) kinetics were assessed using a multispectral optoacoustic imaging system (Vevo LAZR-X, FUJIFILM VisualSonics, Toronto, ON, Canada). Following intravenous administration of ICG (1 mg/kg), dynamic imaging was continuously performed for 500 s to characterize hepatic uptake and clearance.

Liver ROIs were manually defined, and signal enhancement was normalized to baseline values. Longitudinal photoacoustic signal profiles were analyzed using a restricted maximum likelihood (REML)-based mixed-effects model with time treated as a repeated factor and experimental group as a fixed effect.

Quantitative pharmacokinetic parameters, including area under the curve (AUC_0–500_ s), peak signal intensity (Peak Y), and time to peak enhancement (Tmax), were extracted from individual time–signal curves. AUC and Tmax were compared using the Kruskal–Wallis test followed by Dunn’s multiple-comparison test because normality assumptions were not consistently satisfied. Peak Y was analyzed using one-way analysis of variance (ANOVA) followed by Tukey’s multiple-comparison test.

### 2.3. Serum ICG Clearance Assay

Systemic liver clearance was evaluated using a serum ICG retention assay. Following intravenous administration of ICG (1 mg/kg), blood samples were collected via the retro-orbital venous plexus at 0, 300, and 600 s.

The serum ICG concentration immediately after injection (0 s) was defined as 100%, and retention at subsequent time points was expressed as a percentage of baseline. Serum ICG retention at 600 s served as the primary endpoint reflecting systemic hepatic clearance and liver functional reserve.

### 2.4. Histological and Molecular Analyses

In vivo hepatic microarchitecture was evaluated using probe-based confocal laser endomicroscopy after intravenous administration of fluorescein isothiocyanate-dextran.

Following imaging, liver tissues were harvested for histological and molecular analyses. Total RNA was extracted using TRIzol reagent, and quantitative reverse-transcription PCR was performed using SYBR Green chemistry. Relative expression of *Slco1b2* and Abcc2 was calculated using the 2^−ΔΔCt^ method with β-actin as the internal reference.

For immunoblotting, liver lysates were separated by SDS-PAGE and transferred to polyvinylidene fluoride membranes. Primary antibodies included anti-MRP2/ABCC2 (bs-1092R, Bioss, Woburn, MA, USA) and anti-Oatp4 (sc-376904, Santa Cruz Biotechnology, Dallas, TX, USA), which recognizes mouse Oatp1b2. β-Actin served as the loading control. Protein band intensities were quantified using ImageJ (version 1.54f). Molecular analyses were performed in a representative subset of animals (n = 3 per group).

Histological evaluation included Masson’s trichrome staining of paraffin-embedded liver sections and Oil Red O staining of frozen sections. All histological assessments were performed in a blinded manner.

### 2.5. Statistical Analysis

Statistical analyses were performed using GraphPad Prism (version 10.1.2). Data normality was evaluated using the Kolmogorov–Smirnov test. Parametric or nonparametric statistical methods were selected according to data distribution.

Longitudinal MRI and optoacoustic imaging data were analyzed using restricted maximum likelihood (REML)-based mixed-effects models. Intergroup comparisons were performed using one-way ANOVA or Kruskal–Wallis tests with appropriate multiple-comparison corrections.

Associations between MRI-derived parameters, optoacoustic kinetic parameters, serum ICG retention, and hepatocellular transporter expression were evaluated using Spearman rank correlation analysis because several variables did not satisfy normality assumptions. Correlation strength was interpreted according to the classification proposed by Evans, with absolute correlation coefficients (|ρ|) of <0.20, 0.20–0.39, 0.40–0.59, 0.60–0.79, and ≥0.80 indicating very weak, weak, moderate, strong, and very strong correlations, respectively. The direction of the association was determined by the sign of the correlation coefficient, with positive values indicating direct associations and negative values indicating inverse associations.

Data are presented as mean ± standard deviation (SD). All statistical tests were two-sided, and *p* < 0.05 was considered statistically significant.

### 2.6. Safety Statement

No unexpected or unusually high hazards were encountered.

## 3. Results

**Figure 1:**
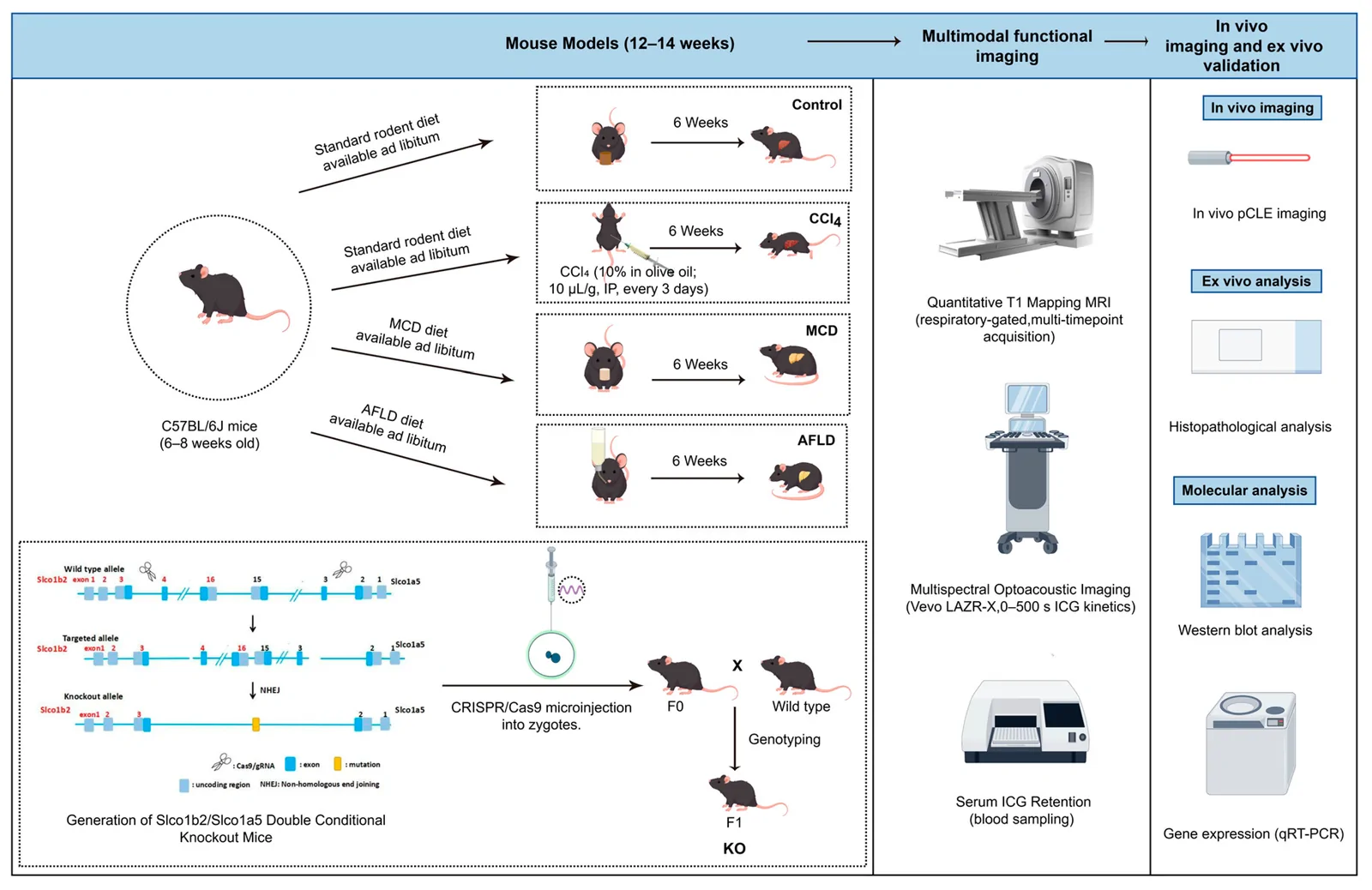
**Schematic overview of the experimental design and multimodal evaluation framework.** The schematic illustration consists of three panels. (**Left**): establishment of experimental liver injury models, including normal control mice (Control), carbon tetrachloride-induced liver fibrosis (CCl_4_), methionine–choline-deficient (MCD) diet-induced steatohepatitis, alcoholic fatty liver disease induced by a Lieber–DeCarli ethanol liquid diet (AFLD), and *Slco1b2*/*Slco1a5* double-knockout mice (KO). (**Middle**): in vivo functional assessment using multimodal imaging approaches, including gadolinium ethoxybenzyl diethylenetriamine pentaacetic acid (Gd-EOB-DTPA)-enhanced magnetic resonance imaging (MRI), indocyanine green (ICG) clearance, and photoacoustic (PA) imaging. (**Right**): in vivo imaging-based and ex vivo validation analyses, including probe-based confocal laser endomicroscopy (pCLE), Western blotting, quantitative PCR (qPCR), and histopathological evaluation. Figure created with Figdraw **(Home for Researchers, Hangzhou, China; https://www.figdraw.com)**.

As summarized in Figure 1, four experimental liver injury models were established alongside normal controls and a genetic negative control, including a transporter-deficient *Slco1b2*/*Slco1a5* double-knockout (KO) model, a carbon tetrachloride-induced fibrosis model (CCl_4_), a methionine–choline-deficient (MCD) diet-induced metabolic injury model, and an alcohol-associated fatty liver disease (AFLD) model. These models represent distinct etiologies of hepatocellular dysfunction and were evaluated using quantitative MRI, optoacoustic imaging, and ex vivo analyses.

**Figure 2:**
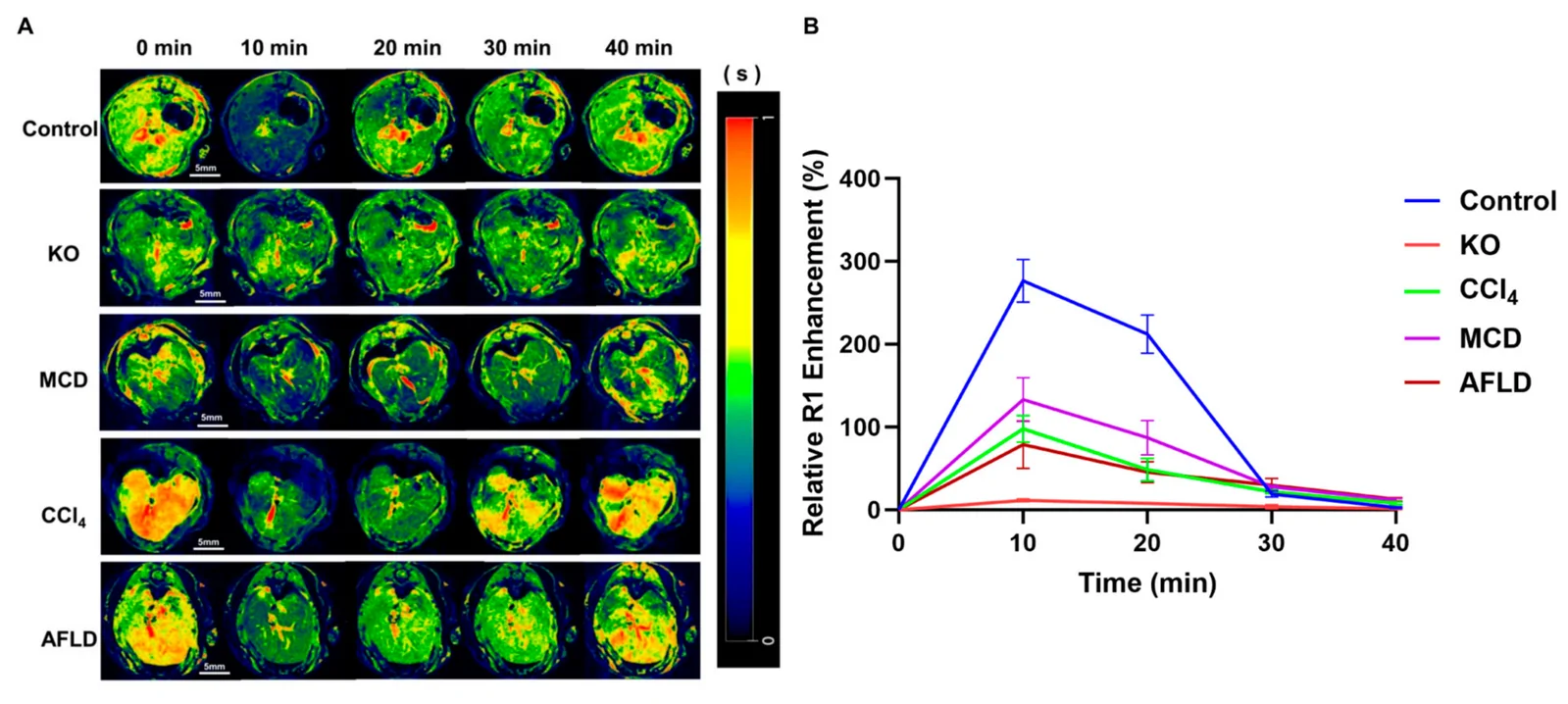
**Hepatic T1 Dynamics and Derived ΔR1% Following Gadoxetic Acid Administration.** (**A**) Representative hepatic T1 parametric maps acquired at baseline and at 10–40 min after contrast administration in each group (unit: seconds). Parametric maps were displayed using a color-coded scale with a fixed range of 0–1 s, enabling direct visual comparison across groups and time points. (**B**) Longitudinal changes in hepatic ΔR1% relative to baseline following Gd-EOB-DTPA administration (mean ± SD, n = 6 per group). All groups exhibited peak enhancement at 10 min, followed by progressive washout over time. The control group demonstrated the greatest enhancement magnitude, whereas the transporter-deficient KO group showed minimal signal increase throughout the dynamic phase. The CCl_4_, MCD, and AFLD models exhibited distinct intermediate enhancement kinetics, reflecting differential impairment of hepatobiliary contrast uptake and retention.

Dynamic changes in hepatic R1 and derived ΔR1% following Gd-EOB-DTPA administration revealed distinct, model-dependent hepatobiliary enhancement kinetics (Figure 2). Representative T1 maps (Figure 2A) were reconstructed using a fixed and explicitly defined color scale (0–1 s) applied uniformly across all groups and time points, enabling direct visual comparison independent of display windowing. The longitudinal absolute hepatic T1 time courses and corresponding quantitative T1 values are provided in Supporting Information Figure S2 and Table S3, respectively, whereas the quantitative ΔR1% values underlying Figure 2B are summarized in Supporting Information Table S4. Baseline hepatic T1 values were comparable across groups, whereas post-contrast reductions varied substantially, consistent with differences in hepatobiliary contrast uptake and retention.

Longitudinal ΔR1% analysis using a restricted maximum likelihood (REML)-based mixed-effects model (Figure 2B) demonstrated significant effects of time (F(1.200, 30.01) = 696.7, *p* < 0.0001), group (F(4, 25) = 125.7, *p* < 0.0001), and time × group interaction (F(16, 100) = 123.4, *p* < 0.0001). Deviation from sphericity (Geisser–Greenhouse ε = 0.3001) was accounted for, and a significant matching effect (χ^2^ = 11.12, *p* = 0.0009) confirmed within-subject correlation. Random-effects estimates indicated moderate inter-individual variability (subject SD = 6.17) relative to residual variability (SD = 10.79).

Kinetic parameterization further differentiated group-specific enhancement patterns. The control group exhibited the highest cumulative enhancement (total area: 5088.67 ± 498.03), reaching a peak ΔR1% of 276.37 ± 25.73 at 10 min. In contrast, the KO group demonstrated minimal enhancement (total area: 238.1 ± 37.73; peak: 11.34 ± 1.86%), indicating severely impaired hepatocellular uptake. The CCl_4_ group showed markedly reduced enhancement (total area: 1722.33 ± 300.68; peak: 97.81 ± 16.14%) compared with controls. The MCD and AFLD groups exhibited intermediate enhancement patterns, with total areas of 2540.67 ± 456.93 and 1604 ± 458.65, and peak values of 133.05 ± 26.28 and 78.7 ± 28.65, respectively. All injury models reached peak enhancement at 10 min, consistent with the control group.

**Figure 3:**
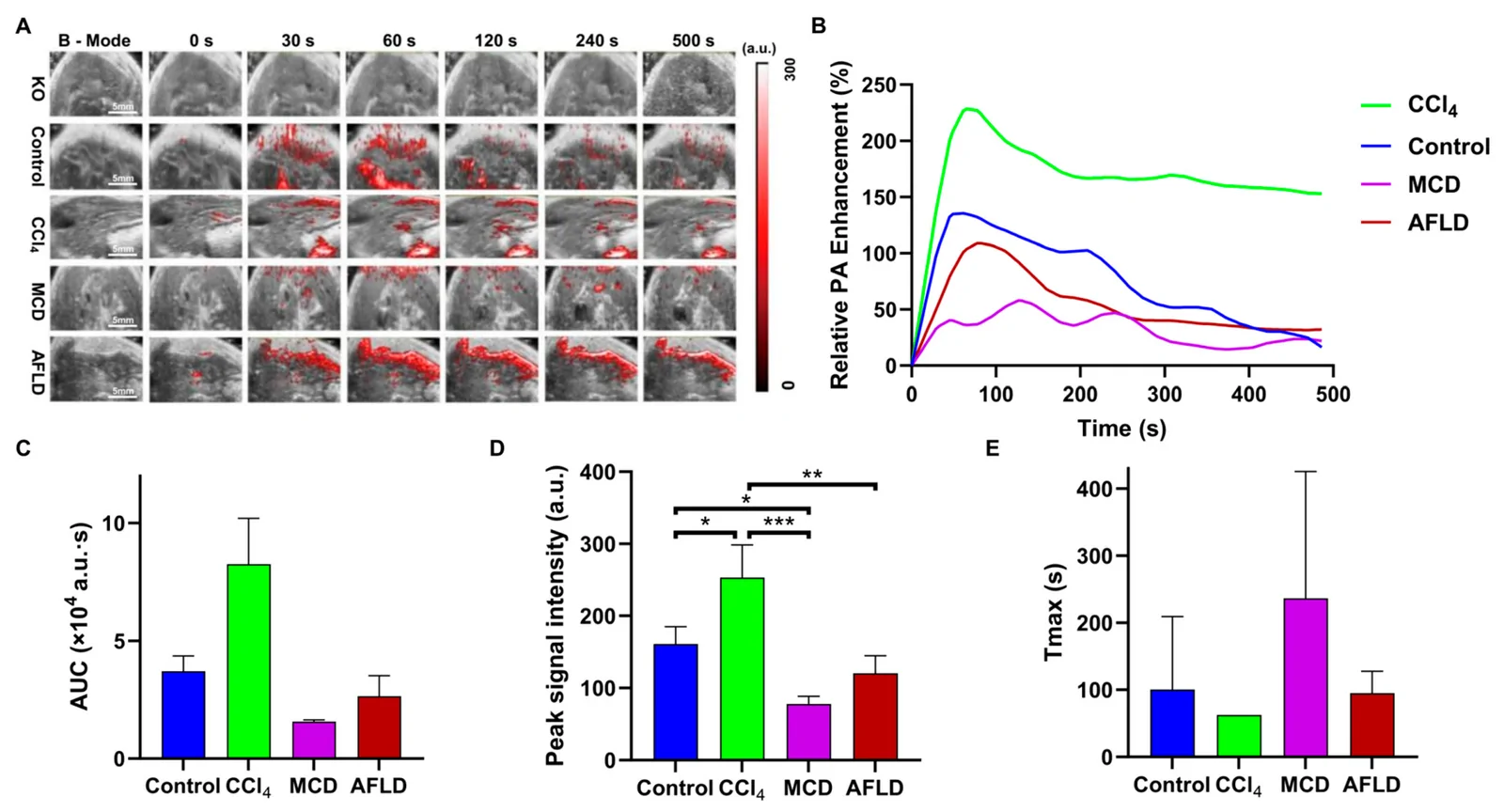
**Hepatic indocyanine green (ICG) pharmacokinetics assessed by multispectral optoacoustic imaging.** Hepatic ICG dynamics were evaluated using a small-animal multispectral optoacoustic imaging system (Vevo LAZR-X, FUJIFILM VisualSonics). (**A**) Representative optoacoustic images acquired before and after intravenous ICG administration, including B-mode reference images and dynamic images at 0, 30, 60, 120, 240, and 500 s. All images are displayed using a fixed color scale (0–300 a.u.) to ensure direct visual comparability across groups and time points. Signal intensity is visualized using a pseudocolor scale ranging from dark (minimum) to bright (maximum), with increasing intensity represented by red-dominant coloration. (**B**) Real-time hepatic ICG enhancement curves over 0–500 s. Curves were smoothed using locally weighted scatterplot smoothing (LOWESS). Enhancement profiles differed significantly over time and among groups (mixed-effects model: time effect, *p* < 0.0001; group effect, *p* = 0.0004). (**C**) Area under the curve (AUC_0–500 s_) of hepatic ICG enhancement. (**D**) Peak signal intensity. (**E**) Time to peak (T_max_). Data are presented as mean ± SD. The KO group was excluded from quantitative kinetic analysis due to negligible hepatic enhancement precluding reliable fitting. *, **, and *** indicate statistical significance at ***p*** **< 0.05,** ***p*** **< 0.01, and** ***p*** **< 0.001**.

Multispectral optoacoustic imaging revealed distinct intrahepatic indocyanine green (ICG) pharmacokinetics across experimental groups (Figure 3A,B). Because the KO group exhibited negligible hepatic signal without a discernible peak, pharmacokinetic fitting was not feasible; analyses were therefore restricted to the Control, CCl_4_, MCD, and AFLD groups (n = 6 per group). Mixed-effects modeling demonstrated significant effects of time (F(7.359, 147.2) = 53.47, *p* < 0.0001), group (F(3, 20) = 75.51, *p* < 0.0001), and time × group interaction (F(90, 600) = 5.963, *p* < 0.0001), with a significant matching effect (χ^2^ = 164.8, *p* < 0.0001). Quantitative AUC_0_–_500_ s differed significantly among groups (*p* = 0.0001), with the highest values observed in the CCl_4_ group and the lowest in the MCD group. Peak signal intensity also differed significantly (*p* < 0.0001), whereas Tmax did not reach statistical significance (*p* = 0.0596), reflecting increased variability in metabolically driven injury models.

**Figure 4:**
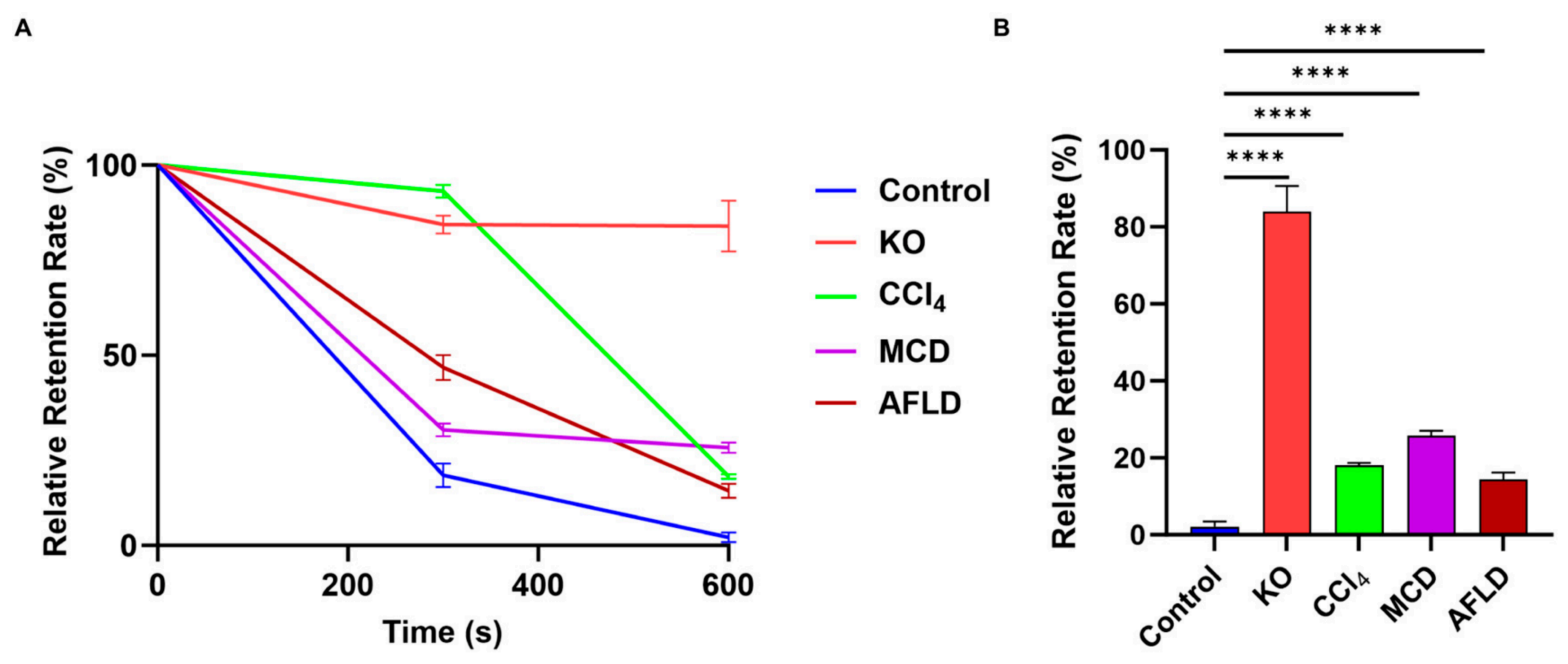
**Serum ICG retention profiles across experimental groups.** (**A**) Time-dependent changes in serum ICG retention following intravenous injection, expressed as a percentage of baseline (0 s). (**B**) Serum ICG retention at 600 s. All non-control groups exhibited significantly higher retention than controls (*p* < 0.0001). Data are presented as mean ± SD. **** indicate statistical significance at ***p*** **< 0.0001**.

Serum ICG retention (Figure 4A) demonstrated distinct systemic clearance profiles. Mixed-effects analysis showed significant effects of time (F(1.30, 48.71) = 6952, *p* < 0.0001), group (F(4, 75) = 1176, *p* < 0.0001), and time × group interaction (F(8, 75) = 538.3, *p* < 0.0001). At 300 s, retention was lowest in controls (18 ± 5.15%) and elevated in the KO and CCl_4_ groups. At 600 s, near-complete clearance was observed in controls (2 ± 1.27%), whereas the KO group remained markedly elevated (84 ± 6.69%), indicating severely impaired systemic elimination. The CCl_4_, MCD, and AFLD groups exhibited intermediate retention levels.

**Figure 5:**
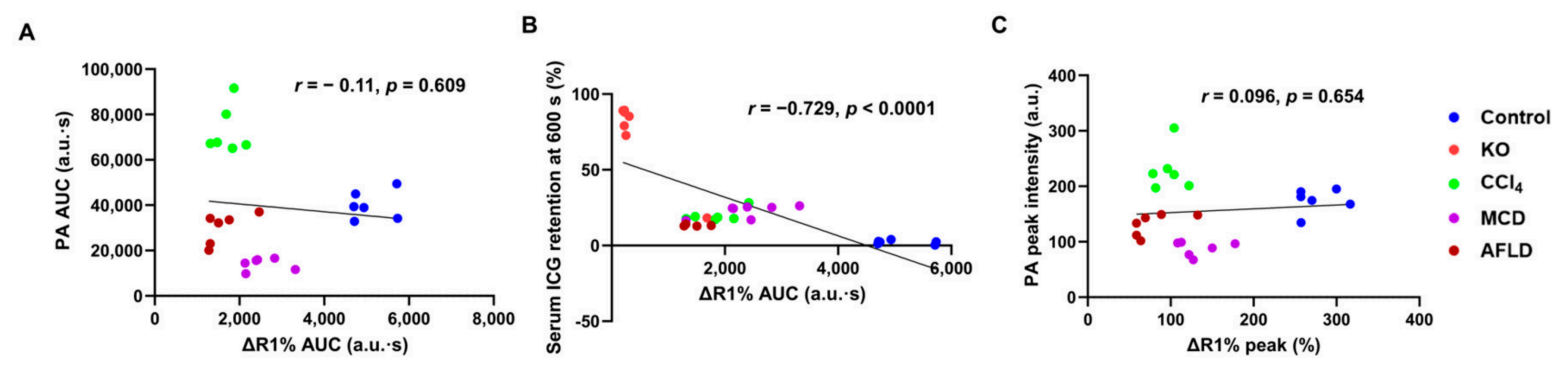
**Correlation analyses between MRI-derived ΔR1% parameters, optoacoustic ICG metrics, and serum ICG retention.** (**A**) Correlation between ΔR1% AUC and optoacoustic AUC (Spearman r = −0.11, *p* = 0.609). (**B**) Correlation between ΔR1% AUC and serum ICG retention at 600 s (Spearman r = −0.729, *p* < 0.0001). (**C**) Correlation between ΔR1% peak and optoacoustic peak intensity (Spearman r = 0.096, *p* = 0.654). The *Slco1b2*/*Slco1a5* double-knockout (KO) mice were excluded only from analyses involving optoacoustic parameters (**A**,**C**) because the absence of effective hepatocellular ICG uptake resulted in negligible hepatic optoacoustic signal intensity, preventing reliable extraction of quantitative kinetic parameters. In contrast, serum ICG retention (**B**) reflects systemic clearance and remained measurable in KO mice; therefore, these animals were retained in the serum ICG analysis. Regression lines are shown solely to facilitate visualization of the overall direction of the associations (positive or negative) and were not used for statistical inference. Correlations were evaluated using Spearman rank analysis.

According to the predefined Evans classification, ΔR1% AUC demonstrated a strong negative correlation with serum ICG retention at 600 s (Spearman’s ρ = −0.729, *p* < 0.0001, n = 30). In contrast, correlations between MRI-derived ΔR1% AUC and optoacoustic AUC (ρ = −0.110, *p* = 0.609, n = 24) and between MRI-derived peak ΔR1% and optoacoustic peak intensity (ρ = 0.096, *p* = 0.654, n = 24) were classified as very weak and were not statistically significant (Figure 5).

**Figure 6:**
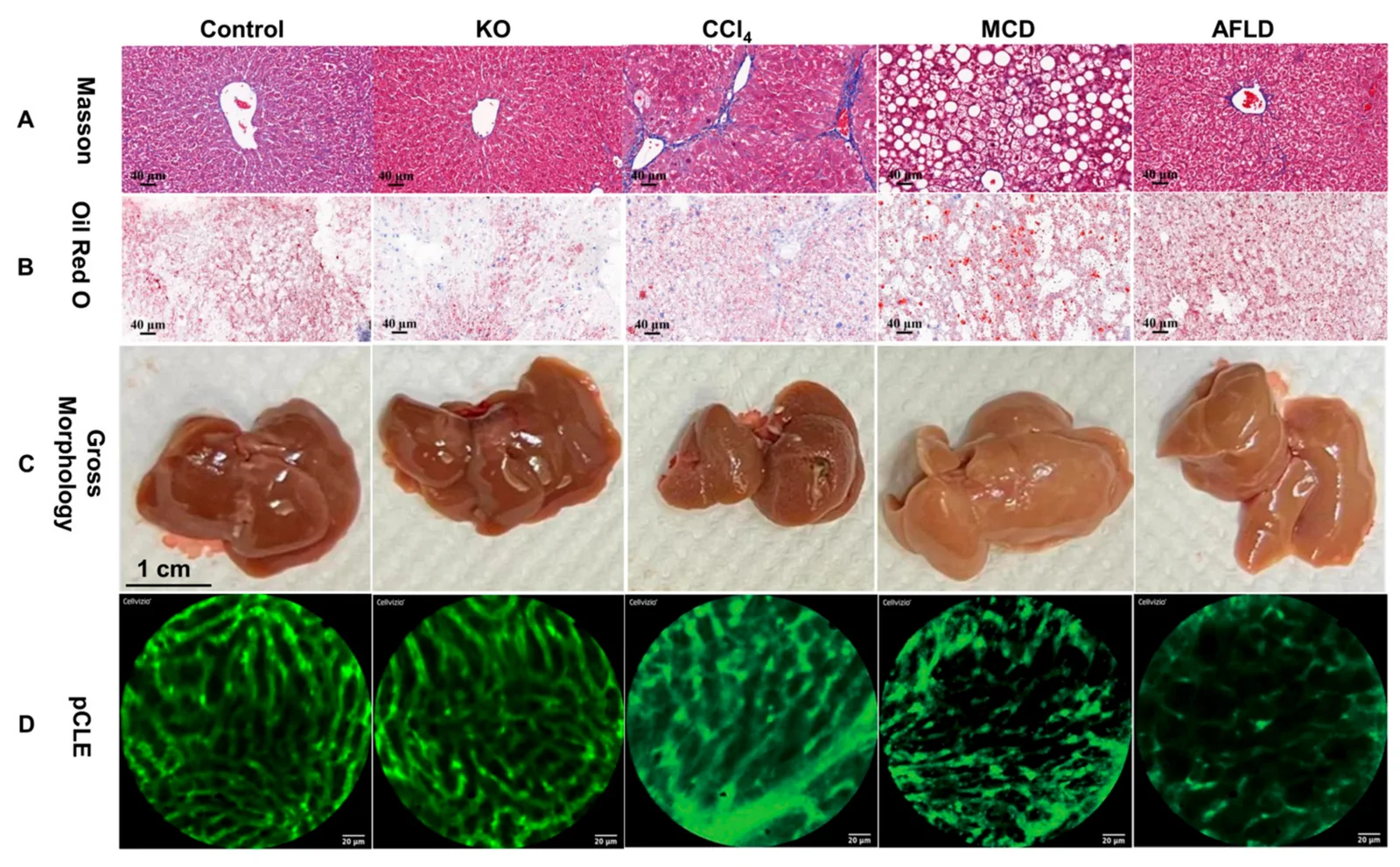
**Histological and in vivo imaging validation of hepatocellular injury across experimental liver models.** (**D**) Representative in vivo probe-based confocal laser endomicroscopy (pCLE) images acquired 10 min after intravenous administration of 70 kDa FITC–dextran. Control and KO groups show preserved sinusoidal architecture, whereas CCl_4_-treated livers exhibit distorted and thickened sinusoids; MCD livers display fragmented sinusoidal structures; AFLD livers show prominent signal-void regions. (**C**) Representative gross liver morphology. (**A**) Masson’s trichrome staining showing collagen deposition (blue). (**B**) Oil Red O staining showing hepatic lipid accumulation (red). Scale bars: (**D**) 20 µm; (**C**) 1 cm; (**A**,**B**) 40 µm. Data are presented as mean ± SD. Statistical analysis was performed using one-way ANOVA followed by post hoc multiple comparisons.

Probe-based confocal laser endomicroscopy (Figure 6A) demonstrated preserved sinusoidal architecture in control and KO groups, whereas CCl_4_-treated livers exhibited marked architectural distortion. Histological analysis (Figure 6C,D) confirmed extensive fibrosis in the CCl_4_ model and pronounced lipid accumulation in the MCD and AFLD models.

**Figure 7:**
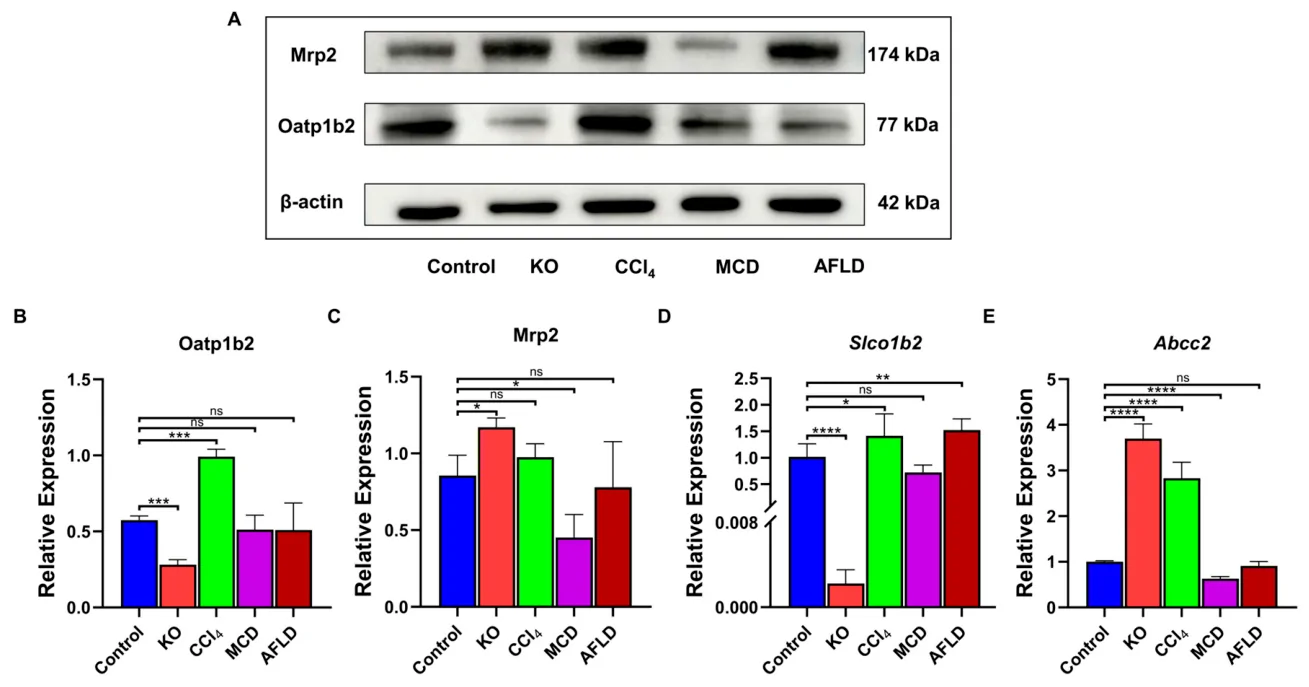
**Hepatic expression of membrane transporters across experimental groups.** (**A**) Representative Western blot images showing protein expression of organic anion-transporting polypeptide 1b2 (Oatp1b2) and multidrug resistance-associated protein 2 (Mrp2) in liver tissue. β-Actin was used as a loading control. (**B**,**C**) Densitometric quantification of Oatp1b2 and Mrp2 normalized to β-actin (n = 3 per group). (**D**,**E**) Relative mRNA expression levels of *Slco1b2* and Abcc2 determined by quantitative real-time PCR (qRT-PCR) and normalized to the reference gene (n = 3 per group). *, **, ***, and **** indicate statistical significance at ***p*** **< 0.05,** ***p*** **< 0.01,** ***p*** **< 0.001, and** ***p*** **< 0.0001**.

Hepatocellular transporter expression showed heterogeneous regulation across models (Figure 7A–C). Western blot analysis (Figure 7A) demonstrated reduced Oatp1b2 expression in the KO group and increased expression in the CCl_4_ model, whereas Mrp2 exhibited an opposing trend. Quantitative transcriptional analysis (Figure 7B) and densitometric quantification (Figure 7C) supported these findings. Notably, residual Oatp1b2-related protein signals were detected in the KO group despite confirmed genomic knockout by PCR genotyping (Supporting Information, Figure S1). However, this residual signal did not translate into functional recovery, as evidenced by minimal ΔR1% response and negligible hepatic ICG uptake, supporting the classification of this model as a functionally transporter-deficient phenotype. Across models, transporter expression patterns did not parallel ΔR1% or ICG-derived functional measurements.

## 4. Discussion

The present study demonstrates that ΔR1% derived from Gd-EOB-DTPA-enhanced T1 mapping provides a quantitative imaging biomarker of hepatic functional reserve across multiple mechanistically distinct liver injury models. Despite marked heterogeneity in histopathology, transporter expression, and enhancement kinetics, ΔR1% consistently differentiated the severity of functional impairment and showed a strong inverse association with systemic ICG retention. These findings suggest that quantitative T1 mapping captures the integrated physiological consequences of hepatobiliary transport dysfunction, indicating that ΔR1% reflects the cumulative functional output of multiple interacting biological processes rather than any single pathological alteration.

Compared with conventional signal intensity-based hepatobiliary MRI, quantitative T1 mapping measures an intrinsic tissue relaxation parameter that is less dependent on scanner settings, pulse sequence parameters, and image windowing, thereby improving reproducibility across imaging systems [33,34,35,36,37]. Expressing enhancement as ΔR1% further normalizes contrast-induced relaxation changes, facilitating longitudinal comparisons under standardized imaging conditions. The enhancement kinetics observed in the present study support the physiological basis of this approach [38,39]. Although all groups reached peak enhancement at approximately 10 min after Gd-EOB-DTPA administration, enhancement magnitude differed markedly among disease models, indicating that liver injury primarily impaired the efficiency of hepatocellular uptake and intracellular retention rather than vascular delivery of contrast. This finding is biologically plausible because Gd-EOB-DTPA is rapidly delivered to the liver through the circulation, whereas its subsequent intracellular accumulation depends on intact hepatocyte transport and preservation of tissue architecture. The almost complete loss of enhancement in the transporter-deficient KO mice and the intermediate enhancement observed in the CCl_4_, MCD, and AFLD models further indicate that ΔR1% reflects the cumulative functional consequences of transporter dysfunction, hepatocyte injury, altered perfusion, and architectural remodeling.

Although both Gd-EOB-DTPA and indocyanine green (ICG) depend on hepatobiliary transport, they interrogate different aspects of liver physiology. Gd-EOB-DTPA-enhanced T1 mapping primarily reflects transporter-mediated hepatocellular uptake and intracellular retention within liver tissue, whereas systemic ICG clearance reflects the integrated whole-organ elimination process involving hepatic perfusion, sinusoidal exchange, plasma protein binding, hepatocyte uptake, intracellular transport, and biliary excretion. Accordingly, MRI-derived ΔR1% and ICG measurements should be regarded as complementary rather than interchangeable functional assessments because they quantify different physiological steps within the hepatobiliary transport pathway. This complementary relationship is illustrated by the correlation analyses shown in Figure 5. Specifically, ΔR1% AUC showed a strong inverse correlation with systemic ICG retention (Figure 5B), whereas no significant correlation was observed between MRI-derived ΔR1% and regional optoacoustic ICG parameters (Figure 5A,C). The lack of a direct correlation between regional optoacoustic ICG kinetics and MRI-derived ΔR1% is therefore biologically expected rather than contradictory, because regional optoacoustic imaging reflects localized intrahepatic signal dynamics whereas MRI-derived ΔR1% and systemic ICG retention represent tissue-level contrast accumulation and global hepatic clearance, respectively. This interpretation is consistent with previous experimental studies and recent meta-analyses showing only moderate correlations between Gd-EOB-DTPA enhancement and ICG clearance, indicating that the two techniques evaluate related but non-identical physiological processes [40,41,42].

An important issue raised by the present study is the interpretation of ΔR1% as a quantitative imaging biomarker. Unlike absolute ΔR1, ΔR1% normalizes the contrast-induced relaxation change to the baseline R1_0_, thereby reducing inter-scan variability and improving robustness for longitudinal studies performed under identical imaging conditions [43,44,45]. However, ΔR1 and ΔR1% represent different biological quantities. Under the fast-exchange assumption, absolute ΔR1 remains approximately proportional to tissue gadolinium concentration, whereas ΔR1% is additionally influenced by baseline tissue relaxation. Because baseline R1_0_ varies with fibrosis, steatosis, edema, inflammation, iron deposition, and other pathological changes, identical intracellular gadolinium concentrations may produce different ΔR1% values in tissues with different baseline relaxation characteristics [43]. Consequently, ΔR1% should be interpreted as a normalized functional index rather than a direct measure of intracellular contrast concentration. It should therefore complement, rather than replace, conventional relaxometric parameters such as absolute R1 or ΔR1 [46,47,48]. In the present study, ΔR1% was selected because the primary objective was to compare hepatic functional reserve across heterogeneous disease models acquired under identical imaging conditions rather than to estimate absolute intracellular gadolinium concentration. Under these conditions, normalization improves robustness while preserving sensitivity to biologically meaningful functional differences.

The strong inverse association between ΔR1% AUC and systemic ICG retention further supports the biological relevance of MRI-derived functional measurements. Because systemic ICG retention reflects whole-organ hepatic clearance, the close relationship observed in this study suggests that ΔR1% captures organ-level functional reserve rather than merely local tissue characteristics. The weaker association between regional optoacoustic parameters and systemic ICG retention further supports this interpretation. Moreover, the overall direction of the observed MRI–ICG relationship agrees with previous experimental and clinical studies, although the reported correlation strength varies among disease etiologies owing to differences in transporter activity, hepatic perfusion, fibrosis, and imaging methodology [49]. This inverse relationship is biologically reasonable because impaired hepatocellular uptake of Gd-EOB-DTPA reduces ΔR1%, while impaired hepatic clearance simultaneously increases systemic ICG retention. Although these measurements reflect different physiological processes, both ultimately depend on preserved hepatobiliary transport capacity.

The apparent dissociation between transporter expression and imaging-derived function suggests that effective hepatobiliary transport is determined by transporter activity rather than protein abundance alone [50,51,52,53]. Increased Oatp1b2 expression in the CCl_4_ model may represent a compensatory response to chronic injury, whereas persistent fibrosis, sinusoidal capillarization, and impaired membrane localization likely limit effective substrate transport despite increased protein expression. Likewise, the residual Oatp1b2-related immunoreactivity detected in the KO mice probably reflects antibody cross-reactivity or expression of homologous transporters that do not restore functional uptake.

Several limitations should be acknowledged. First, transporter characterization was limited to expression analyses without direct assessment of membrane localization or transport activity. Second, enhancement kinetics were summarized using peak enhancement and AUC rather than formal pharmacokinetic modeling. Third, quantitative T1 mapping remains susceptible to acquisition protocol, respiratory motion, and fitting methodology, emphasizing the need for standardized imaging protocols. Therefore, the diagnostic performance and clinical utility of ΔR1% should be confirmed in prospective studies involving patients with diverse etiologies and stages of chronic liver disease.

Future studies should validate ΔR1% in larger prospective clinical cohorts, integrate quantitative T1 mapping with pharmacokinetic modeling and direct measurements of transporter function, and establish standardized acquisition and analysis protocols for multicenter implementation. Such studies will be essential for defining clinically applicable quantitative thresholds and facilitating translation of transporter-mediated T1 mapping into routine assessment of hepatic functional reserve.

## 5. Conclusions

This study establishes a quantitative transporter-mediated imaging framework based on Gd-EOB-DTPA-enhanced T1 mapping and demonstrates that ΔR1% robustly differentiates hepatic functional reserve across multiple mechanistically distinct liver injury models. Unlike conventional signal intensity measurements, ΔR1% provides a standardized quantitative imaging biomarker that integrates hepatobiliary transport dysfunction while complementing, rather than replacing, established liver function tests. These findings provide mechanistic evidence supporting the clinical translation of quantitative T1 mapping as a noninvasive imaging strategy for assessing hepatic functional reserve.

## Data Availability

The original contributions presented in this study are included in the article/Supplementary Material. Further inquiries can be directed to the corresponding author.

## Supplementary Materials

The following supporting information can be downloaded at: https://www.mdpi.com/article/10.3390/biomedicines14081695/s1. Table S1: PCR Primers, Reaction System, and Cycling Conditions; Table S2: MRI Acquisition Parameters for Quantitative T1 Mapping; Table S3: Baseline and Post-Contrast Hepatic T1 Values; Table S4: Quantitative Relative R1 Enhancement (ΔR1%) Following Gd-EOB-DTPA Administration; Figure S1: PCR Genotyping of Knockout Mice; Figure S2: Longitudinal hepatic T1 values measured at baseline.

## Abbreviations

Gd-EOB-DTPA	Gadolinium ethoxybenzyl diethylenetriamine pentaacetic acid (gadoxetate disodium), a hepatocyte-specific magnetic resonance imaging (MRI) contrast agent.
MRI	Magnetic Resonance Imaging
ΔR1%	Change in longitudinal relaxation rate (R1) before and after contrast administration
ICG	Indocyanine Green
LFR	Liver Functional Reserve
CCl_4_	Carbon Tetrachloride (used to induce liver fibrosis)
MCD	Methionine-Choline Deficient diet (model of non-alcoholic steatohepatitis)
AFLD	Alcoholic Fatty Liver Disease (Lieber-DeCarli diet model)
KO	Knockout (*Slco1b2*/*Slco1a5* double-knockout mice)
Oatp	Organic anion transporting polypeptides; murine orthologs of human OATP transporters.
Mrp	Multidrug resistance-associated proteins, key hepatocellular efflux transporters.
pCLE	Probe-based Confocal Laser Endomicroscopy
qRT-PCR	Quantitative Reverse-Transcription Polymerase Chain Reaction (qRT-PCR)
ANOVA	Analysis of Variance
SD	Standard Deviation
TBST	Tris-Buffered Saline with Tween (washing buffer)
HRP	Horseradish Peroxidase (enzyme for antibody conjugation)
ECL	Enhanced Chemiluminescence (for blot detection)
ROI	Region of Interest
RE	Relative Enhancement (in MRI signal intensity)
MELD score	Model for End-Stage Liver Disease score
FITC-dextran	Fluorescein Isothiocyanate-labeled dextran (vascular tracer)
