# Supplementary material for: Gadoxetic Acid-Enhanced T1 Mapping Enables Transporter-Mediated Molecular Imaging of Liver Functional Reserve
Source: Biomedicines. 2026 Jul 28;14(8):1695. doi: 10.3390/biomedicines14081695 (PMC13509626; doi:10.3390/biomedicines14081695)
Supplement: Supplementary file 1 [file biomedicines-14-01695-s001.zip › biomedicines-4346806-supplementary.pdf]

Section S1. Genotyping Methods

Mouse Tail Genomic DNA Extraction

Genomic DNA was extracted from mouse tail biopsies using a proteinase K digestion–ethanol precipitation method. Approximately 0.3–0.5 cm of tail tissue was collected from mice older than 2 weeks of age (adjusted according to tail size to ensure comparable tissue volume) and transferred into labeled 1.5 mL microcentrifuge tubes.

Samples were incubated in 0.5 mL mouse tail lysis buffer (GenePool, Cat# GPQ19388) supplemented with 50 μL proteinase K and digested at 56 °C overnight with gentle agitation to ensure complete lysis. The lysates were centrifuged at 12,000 rpm for 10 min at room temperature.

The supernatant was transferred to a fresh tube, followed by the addition of 1 mL absolute ethanol. After inversion to precipitate DNA, samples were centrifuged at 13,000 rpm for 15 min at 4 °C, and the supernatant was discarded.

The DNA pellet was washed with 75% ethanol, centrifuged, briefly air-dried, and dissolved in 80–100 μL TE buffer (pH 8.0) or nuclease-free water.

PCR Amplification for Genotyping

PCR amplification was performed using a 2× Taq Master Mix (CWBIO, Cat# CW0682). Primer sequences, reaction components, and cycling conditions are summarized in Table S1.

PCR products were analyzed by agarose gel electrophoresis using a DNA ladder (DM2000, CWBIO) as a molecular weight reference.

Table S1. PCR Primers, Reaction System, and Cycling Conditions

Primer Sequences

Primer	Sequence (5’→3’)	Type
P1	ACTCCCCTAAGTCTGAACCA	Forward
P2	AACTGTGGGTCACTACCCTT	Reverse
P3	TGTTTGAGGGACGCTACAGA	Reverse

PCR Reaction Mixture

Component	Volume (μL)
ddH_2O	7.8
2× Taq Master Mix	10
Primer I	0.4
Primer II	0.4
Primer III	0.4
Genomic DNA	1
**Total**	**20**

Cycling Conditions

Step	Temp (°C)	Time	Note
Initial denaturation	98	2 min	
Denaturation	98	20 s	
Annealing	63	20 s	
Extension	72	1 min	35 cycles
Final extension	72	5 min	
Hold	12	—	

Genotype Interpretation

- Wild type: 598 bp
- Heterozygote: 598 + 812 bp
- Homozygote: 812 bp

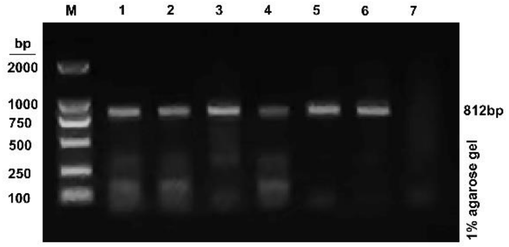

Figure S1. PCR Genotyping of Knockout Mice.

PCR products were generated using a three-primer strategy and analyzed by 1% agarose gel electrophoresis.

- Lane M: DNA marker
- Lanes 1–6: representative samples showing a single 812 bp band (homozygous knockout)
- Lane 7: negative control

The absence of the 598 bp band confirms successful disruption of the wild-type allele.

Section S2. MRI Methodological Details

Table S2. MRI Acquisition Parameters for Quantitative T1 Mapping

Parameter	Value
Scanner	9.4 T Bruker BioSpec (Bruker,

	Ettlingen, Germany)
RF coil	26 mm mouse body volume coil
Sequence	Respiratory-gated VTR-FLASH
T1 mapping strategy	Variable repetition time (VTR) method
Excitation flip angle	15°
Repetition times (TRs)	Multiple TRs acquired under identical imaging geometry
Echo time (TE)	2.0 ms
Field of view (FOV)	30 × 30 mm²
Matrix size	256 × 256
Slice thickness	0.5 mm
In-plane spatial resolution	0.15 × 0.15 mm²
Respiratory gating	Enabled throughout acquisition
Contrast agent	Gd-EOB-DTPA (Primovist®, Bayer Healthcare)
Contrast dose	0.025 mmol/kg
Post-contrast acquisition time points	10, 20, 30, and 40 min
Acquisition time per T1 map	Approximately 4–6 min

Footnote

The acquisition duration varied slightly depending on respiratory gating efficiency and animal respiratory stability during imaging.

Section S3. Additional Quantitative Data

Table S3. Baseline and Post-Contrast Hepatic T1 Values

Group	Baselin	10 min	20 min	30 min	40 min

	e T1 (ms)				
**Control**	732±38	195±18	236±21	616±34	712 ± 37
**KO**	752±33	676±38	697±31	726±44	742 ± 23
**CCl_4**	834±13	424±33	565±50	685±14	773 ± 18
**MCD**	841±27	365±44	455±54	658±24	752 ± 21
**AFLD**	865±17	494±70	599±51	668±39	768 ± 16

Values are presented as mean ± SD (*n* = 6 per group). Baseline represents pre-contrast hepatic T1 values, and subsequent measurements were obtained at 10, 20, 30, and 40 min following intravenous Gd-EOB-DTPA administration.

ΔR1 (%) was calculated as:

$$\Delta R1\% = \frac{R1_{post} - R1_{pre}}{R1_{pre}} \times 100$$

Where R1=1/T1

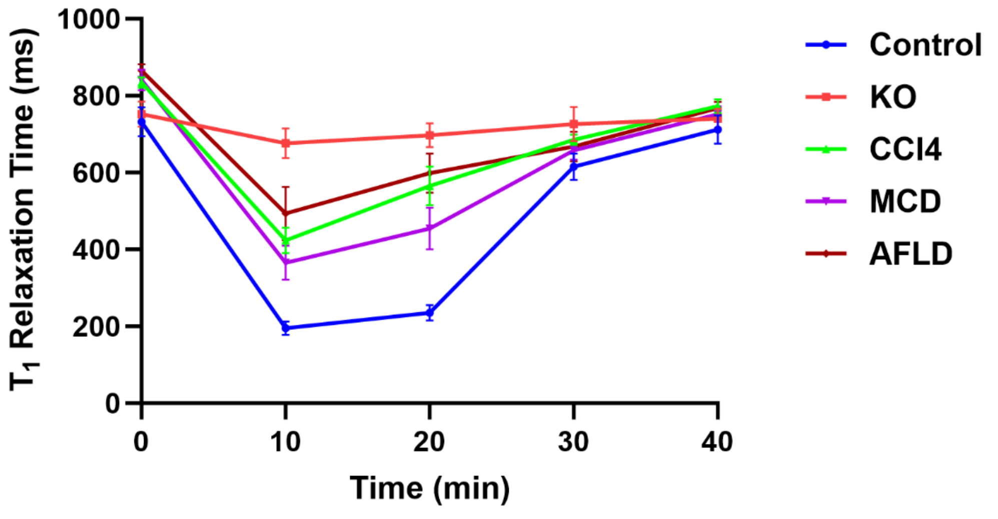

Figure S2. Longitudinal hepatic T1 values measured at baseline (0 min, before Gd-EOB-DTPA administration) and at 10, 20, 30, and 40 min after intravenous Gd-EOB-DTPA administration in the five experimental groups. Absolute hepatic T1 relaxation times (ms) are plotted as a function of time. Data are presented as mean ± SD. In contrast to the normalized ΔR1% parameter shown in the main manuscript, absolute T1 values illustrate the magnitude of longitudinal relaxation changes following contrast administration and facilitate direct comparison of baseline and post-contrast tissue relaxation across experimental groups.

Table S4. Quantitative Relative R1 Enhancement (ΔR1%) Following Gd-EOB-DTPA Administration

Group	10 min	20 min	30 min	40 min
**Control**	276 ± 26	212 ± 23	19 ± 4	3 ± 1
**KO**	11 ± 2	8 ± 1	4 ± 2	2 ± 1
**CCl_4**	98 ± 16	49 ± 14	22 ± 2	8 ± 2
**MCD**	133 ± 26	87 ± 21	28 ± 2	12 ± 2
**AFLD**	79 ± 29	45 ± 13	30 ± 8	13 ± 2

Values are presented as mean ± SD (*n* = 6 per group). Relative R1 enhancement (ΔR1%) was calculated from pre-contrast (baseline) and post-contrast longitudinal relaxation rates according to Equation (1) in the main text. These quantitative values correspond to the longitudinal ΔR1% curves presented in Figure 2B.
